# Molecular screening of the human parvoviruses B19 and bocavirus 1 in the study of congenital diseases as applied to symptomatic pregnant women and children

**DOI:** 10.1099/acmi.0.000037

**Published:** 2019-06-20

**Authors:** Maria Belen Salbetti, Mauro Sebastian Pedranti, Paula Barbero, Paula Molisani, Martina Lazzari, Nicolas Olivera, Maria Beatriz Isa, Ariel Bertoldi, Laura Moreno, Maria Pilar Adamo

**Affiliations:** ^1^ Laboratorio de Rubéola y Parvovirus, Instituto de Virología “Dr J. M. Vanella”, Facultad de Ciencias Médicas, Universidad Nacional de Córdoba, Enf. Gordillo Gómez s/n, Ciudad Universitaria, Córdoba, Argentina; ^2^ Área de Epidemiología, Ministerio de Salud de la Provincia de Córdoba, Av. Vélez Sarsfield 2311, Córdoba, Argentina; ^3^ Clínica Universitaria Reina Fabiola, Córdoba, Oncativo 1248, Córdoba, Argentina; ^4^ Cátedra de Clínica Pediátrica, Hospital Universitario de Maternidad y Neonatología, Facultad de Ciencias Médicas, Universidad Nacional de Córdoba, Rodríguez Peña 285, Córdoba, Argentina

**Keywords:** erythrovirus, bocavirus, pregnancy, newborn, foetal hydrops, abortion

## Abstract

**Introduction:**

B19 virus (B19V) and bocavirus 1 (HBoV1) are human pathogenic parvoviruses that are prevalent worldwide and are responsible for a diverse and not yet fully established spectrum of clinical manifestations.

**Objective:**

To screen B19V and HBoV1 in patients with clinical manifestations associated with acquisition of the infection during gestation.

**Methods:**

A retrospective, observational study was performed that included serum samples from patients without a previous known aetiology. B19V and HBoV1 were determined by end-point PCR. Positive samples were genotyped.

**Results:**

A total of 106 serum samples were analysed, 61 from pregnant women and 45 from neonates and paediatric patients. None were positive for HBoV1, while B19V was detected in 37/106 [34.9 %, 95 % confidence interval (CI): 26.5–44.4] of the samples studied. In the group of pregnant women, 28/61 (45.9 %, 95 % CI: 34.0–58.3) were B19V-positive, and 2 of them had foetal anaemia followed by hydrops and foetal death, 3 were associated with a history of recurrent pregnancy loss and there was 1 case of spontaneous abortion. B19V was also detected in cases of maternal febrile exanthema, polyhydramnios, oligohydramnios and foetal ascites. In the group of children, 9/45 (20.0 %, 95 % CI: 10.9–33.8) neonatal patients were B19V-positive, and this was associated with foetal hydrops, TORCH syndrome and cardiac alterations. The nucleotide sequences analysed confirmed the identity of B19V genotype 1.

**Conclusions:**

We found no evidence to indicate the presence of HBoV1 in maternal blood or in the newborns/paediatric patients (hence providing no support for the supposed vertical transmission). On the other hand, the high frequency of B19V in the pathologies studied indicates the importance of molecular diagnosis in both the mother and the child. Future efforts should contribute to early detection and characterization of infections.

## Introduction

Parvovirus B19 (B19V, *Primate erythroparvovirus 1*) and bocavirus 1 (HBoV1, *Primate bocaparvovirus 1*), both members of the family *Parvoviridae,* are human pathogens that are responsible for various diseases, whose full spectrum has not yet been established [1].

B19V is transmitted by respiratory secretions, vertically from the mother to the foetus and through transfusions and blood products [2]. With a marked tropism for erythroid progenitor cells of the bone marrow and foetal liver, infection produces a high-titre viraemia [3, 4]. As the infected cells die when the viral progeny is released, infection produces a decrease in reticulocytes, haemoglobin and platelets, but it is the immunological and haematological characteristics of the host that determine the clinical manifestations. In immunocompetent individuals without haematological co-morbidity, it does not result in anaemia due to the prolonged half-life of red blood cells and because the immune response limits the infection. They can present with erythematous rash and arthralgia linked to antigen–antibody complexes, but between 20 and 50 % of the cases are asymptomatic. When the clinical manifestations are present, specific IgM and IgG can already be detected, so the presumptive diagnosis in these cases is usually resolved by antibody tests [5–7]. Since the transmission of infection occurs at the peak of viraemia, before the symptoms, these patients (frequently school-aged children) have already transmitted the virus to susceptible contacts by the time the diagnosis is made. When a pregnant woman has symptoms or has been in contact with a patient, there is a risk of intrauterine infection, which can occur several weeks or even months before the onset of marker foetal signs and symptoms. In this situation, a diagnosis based solely on serology in maternal serum may not be reliable. For the diagnosis using foetal or neonate clinical samples, the fact that these patients may lack a typical immune response should be taken into account. Thus viral genome detection is recommended [8].

The destruction of infected foetal cells can trigger severe complications related to anaemia, hydrops, heart failure and foetal death [9, 10], conditions that are included in the TORCH syndrome (a cluster of symptoms caused by congenital toxoplasmosis, rubella, cytomegalovirus, herpes simplex and other organisms, such as syphilis, parvovirus, varicella and Zika virus). A recent survey reported that 71 % of congenital B19V infections ended in spontaneous abortions or stillbirth, while among live births 18 % had hydrops, anaemia or cardiomegaly [11]. In addition, B19V infection during pregnancy has been associated with placental and amniotic fluid defects [12]. The involvement of B19V in foetal pathology may be underdiagnosed and therefore more common than previously believed.

On the other hand, HBoV1 causes acute respiratory infection, mainly in paediatric patients [13–15], but also in adults [16–18]. HBoV1 is not strictly a respiratory virus; it can produce viraemia and has been detected in the enteric tract, even in healthy adults [19]. The natural history of the infection has not been clearly elucidated, but it is known that HBoV1 can establish persistent infections [20, 21] and latent or episomal HBoV1 DNA has been detected in normal and pathological host tissues (tonsils, adenoids, colon) [18, 22–24]. Reinfections without clinical manifestations are also possible [16, 21]. All of this indicates its ability to maintain itself and possibly transmit from asymptomatic adults, and it could also potentially be transmitted through the placenta and cause foetal damage during pregnancy.

The aim of this study was to screen for B19V and HBoV1 in pregnant women and children up to 11 months of age with clinical manifestations of unknown aetiology that might be attributable to parvoviral infection acquired during gestation.

## Methods

### General design

A retrospective, observational study was carried out using samples from unrelated symptomatic pregnant women and newborn/paediatric patients. The study samples were selected from the laboratory biobank, considering the registered associated data of the patients (clinical manifestations that in the presumptive diagnosis could be attributed to parvoviral infection during pregnancy). The samples meeting the inclusion criteria with sufficient sample volume for the required assays were subjected to molecular detection of B19V and HBoV1, sequencing and serological testing for B19V.

### Ethical considerations

The research protocol was approved by an independent Ethics Committee, the Institutional Committee of Ethics in Health Research of the Reina Fabiola University Hospital, Córdoba, Argentina.

### Study population, inclusion/exclusion criteria and data

The selection criteria were as follows.

1. Symptomatic pregnant women: pregnant patients of any age and gestation period with one or more signs/symptoms suggesting parvovirus infection: fever, rash, arthritis, arthralgia, anaemia, spontaneous abortion of unknown aetiology, antecedents of recurrent abortion, and anomalies detected in the product of conception during ultrasound controls, such as hydrops, foetal anaemia, oligohydramnios, polyhydramnios and other signs of TORCH syndrome, such as intrauterine growth retardation and preterm delivery.
2. Neonatal and paediatric patients: newborns and children up to 11 months of age with signs/symptoms associated with congenital parvovirus infection (conditions acquired during intrauterine development): foetal hydrops, anaemia, hepatitis, myocarditis, coagulation disorders associated with hepatic dysfunction, hepatosplenomegaly, renal failure without previously known aetiology, sepsis and other signs included in TORCH syndrome (manifestations in the spectrum of congenital rubella syndrome (CRS), preterm birth, intrauterine growth retardation, microcephaly, cardiac alterations, hepatomegaly and jaundice at birth).

We excluded samples from patients for whom any other aetiological cause was suspected and confirmed for the pathologies studied (including syphilis, *Toxoplasma* *gondii*, rubella, cytomegalovirus, herpes simplex, varicella-zoster virus and HIV) and samples with insufficient volume for nucleic acid extraction. The mentioned criteria were applied for the selection of registered patients between March 2008 and March 2018.

The clinical and epidemiological data for the patients (date, age, clinical manifestations, presumptive diagnosis, other studies/tests performed and results) that were available from the laboratory database were used in the analysis, and the results obtained are described using frequencies (percentages), proportions, 95 % confidence interval (CI) and odds ratio (OR).

### Molecular screening

Nucleic acids were obtained from a 200 µl aliquot of serum using Axygen (Corning, USA) extraction columns and the AxyPrep Body Fluid Viral DNA and RNA Purification Miniprep kit. The extract was stored in TE buffer at −20 °C until it was used in the PCR assays.

For the detection of B19V, a qualitative PCR technique was applied with primers designed in the laboratory for an NS1 region of 242 bases comprising nucleotides 2035 through 2276 of the genome with GenBank access number NC_000883.2 – forward: 5′CACTATGAAAACTGGGCAATAAAC (nt 2035–2058) and reverse: 5′AATGATTCTCCTGAACTGGTCC (nt 2276–2255) (these primers are located in the conserved regions of the three known genotypes of B19 V). The following reagents were used: 5 % DMSO, 2.5 mM MgCl_2_, 0.2 mM each deoxynucleotide, 0.2 µM each primer, 0.02 U µl^−1^ *Taq* DNA polymerase and the reaction buffer provided with the enzyme. The amplification programme included 35 cycles of denaturation at 94 °C, hybridization at 55 °C and an extension at 72 °C, plus a final extension of 4 min at 72 °C [5, 25]. Negative and positive control samples (10 000 copies ml^−1^ recombinant B19V) were included in each assay.

For the detection of HBoV1, a conventional PCR technique was applied, with primers directed to the region of 354 bases of NP1 comprising nucleotides 2351 through 2704 of the genome with GenBank access number DQ000495.1 – forward: 5′GAGCTCTGTAAGTACTATTAC (nt 2351–2371) and reverse: 5′CTCTGTGTTGACTGAATACAG (nt 2704–2684) [26]. The following reagents were used: 2.5 mM MgCl_2_, 0.2 mM each deoxynucleotide, 0.4 µM each primer (Invitrogen), 0.02 U µl^−1^ *Taq* DNA polymerase (Invitrogen) and the reaction buffer provided with the enzyme. The amplification programme included 35 cycles of denaturation at 94 °C, hybridization at 48 °C and an extension at 72 °C, plus a final extension of 10 min at 72 °C. Negative and positive clinical samples that had previously been confirmed by sequencing [14] were included in each assay as controls. All primers and PCR reagents were provided by Invitrogen/Thermo Fisher Scientific, Argentina.

For the visualization of the PCR products, 5 µl of reaction was separated by electrophoresis in 10 % polyacrylamide gel. The gels were fixed with 20 % ethanol/5% acetic acid, stained with 0.011 M AgNO_3_ and developed with 3 % sodium hydroxide/1% formol (sPAGE).

### Genotyping

For the amplification of the NS1 region of B19V, we designed the primer pairs shown in Table 1, optimizing each fragment protocol by adjusting the annealing temperature. The PCR assays for subsequent sequencing were carried out in a total volume of 50 µl with 5 µl template and the reagents specified previously for the detection of B19V. The cycling protocols included 35 cycles of denaturation at 94 °C, hybridization at the annealing temperatures indicated in Table 1 and an extension at 72 °C, plus a final extension for 4 min at 72 °C. The integrity and quantity of the amplified fragments were corroborated by sPAGE as described above and by quantification of the product using the Qubit fluorometer (Invitrogen) with the reagents provided by the manufacturer. The fragments to be sequenced were purified with Qiagen QIAquick PCR Purification kit and the sequencing was performed bidirectionally (Macrogen, Inc., Republic of Korea) using the ABI PRISM 3100 Genetic Analyzer/BigDye Terminator v. 3.1 (Applied Biosystems).

**Table 1.** Primer pairs for B19V NS1 region amplification

Amplicon	Primers (5′>3′ sequence)		Location (nucleotides)	Size (nucleotides)	*T*_ann_ (°C)
673–1471	F	GCTAACGATAACTGGTGGTGC	673–693	799	51
	R	CCTGCTCAAAGTCTGTATGC	1452–1471		
1355–2038	F	TAAGCAGTAGTCACAGTGGAAGT	1355–1377	684	52
	R	CCCAGCTTTGTGCATTACACC	2018–2038		
1817–2666	F	TGCGTGGAAGTGTAGCTGTG	1817–1836	850	55
	R	CATCACTTTCCCACCATTTGCC	2645–2666		
2545–3298	F	TGCCATGTGGGAGCTTCTAA	2545–2564	754	60
	R	TTCTGAGGCGTTGTAAGCGG	3279–3298		

The obtained sequences were edited and analysed using clustal (www.ebi.ac.uk/Tools/msa/clustalo/) and mega X (www.megasoftware.net), including the following sequences of complete genomes available at GenBank: NC_000883.2, KC013324.1, KC013308.1, AB030694.1, KM393169.1, FJ591158.1, AY504945.1, KR005643.1, AY386330.1, AF162273.1, FN598217.1, FN598218.1, AY044266.2, DQ333426.1, KF724387.1, AB550331.1, AY582125.2, DQ408305.1, DQ234779.2 and AY083234.1. The phylogeny reconstruction was performed by using the neighbour-joining protocol with genetic distance matrices based on the Kimura two-parameter model and 1000 bootstrap replications.

### Serology

When necessary (i.e. for samples without previous determination of B19V-specific IgM and/or IgG), anti-B19V antibodies were determined using enzyme-linked immunosorbent assays (Ridascreen, R-biopharm), following the manufacturer’s instructions. For IgM assay serum, samples were treated with RIDA RF-Absorbens (R-biopharm) for the precipitation of IgG antibodies.

## Results

### Population studied

Serum samples from 106 patients with clinical manifestations potentially associated with parvovirus infection during pregnancy were included. Of these, 61 corresponded to pregnant women and 45 to neonatal or paediatric patients. In the first group, the age range was 16 to 40 years old (average: 26.8±7.0 years) and in the group of neonates and children the patients were between 2 days and 11 months old. The most prevalent pathologies among pregnant patients were foetal hydrops (21/61, 34%), recurrent abortion (12/61, 20%) and polyhydramnios (6/61, 10%), while among newborns and paediatric patients they were foetal hydrops (14/45, 31%), TORCH/suspected CRS (13/45, 28%) and microcephaly (3/45, 13%) (see Table 2).

**Table 2.** Pathologies in all patients studied and in B19V-positive cases

Pathologies/presumptive diagnosis	Cases studied	B19V DNA	Anti-B19V IgM	Anti-B19V IgG
***Pregnant patients***				
Foetal hydrops	21	8	5	17
Foetal ascites	3	[Table-fn tbl2fn2]2	0	[Table-fn tbl2fn2]2
TORCH	[Table-fn tbl2fn3]3	2[Table-fn tbl2fn1]*	[Table-fn tbl2fn1]1	[Table-fn tbl2fn2]2
Anaemia, foetal hydrops and abortion	[Table-fn tbl2fn2]2	2†[Table-fn tbl2fn2]	0	[Table-fn tbl2fn2]2
Spontaneous abortion	[Table-fn tbl2fn2]2	1†	[Table-fn tbl2fn1]1	[Table-fn tbl2fn2]2
Recurrent abortion	12	[Table-fn tbl2fn3]3	[Table-fn tbl2fn1]1	10
Polyhydramnios	6	4	[Table-fn tbl2fn3]3	4
Oligohydramnios	[Table-fn tbl2fn3]3	[Table-fn tbl2fn1]1	0	[Table-fn tbl2fn2]2
Foetal hydrocephalus	[Table-fn tbl2fn2]2	[Table-fn tbl2fn2]2	0	[Table-fn tbl2fn1]1
Dysmorphic lateral ventricles	[Table-fn tbl2fn1]1	0	0	0
Cystic hygroma	1	0	0	[Table-fn tbl2fn1]1
Thrombocytopenia	[Table-fn tbl2fn1]1	0	0	[Table-fn tbl2fn1]1
Recurrent exanthema	[Table-fn tbl2fn1]1	0	0	0
Febrile exanthema/acute B19 infection	[Table-fn tbl2fn3]3	[Table-fn tbl2fn3]3	[Table-fn tbl2fn2]2	[Table-fn tbl2fn3]3
*Total in the group of pregnant patients*	*61/106* (***57.5%***)	*28/61* (***45.9%***)	*13/61* (***21.3%***)	*47/61* (***77.0%***)
***Neonatal and paediatric patients***				
TORCH (suspected CRS, signs not specified)	13	4‡[Table-fn tbl2fn3]	[Table-fn tbl2fn1]1	6
foetal hydrops	14	4	[Table-fn tbl2fn1]1	11
Cardiac alterations	[Table-fn tbl2fn1]1	[Table-fn tbl2fn1]1	0	[Table-fn tbl2fn1]1
Congenital myocarditis	[Table-fn tbl2fn1]1	0	0	[Table-fn tbl2fn1]1
Hepatosplenomegaly	[Table-fn tbl2fn1]1	0	0	[Table-fn tbl2fn1]1
Hepatitis	[Table-fn tbl2fn1]1	0	0	[Table-fn tbl2fn1]1
Liver dysfunction	1	0	0	[Table-fn tbl2fn1]1
Microcephaly	[Table-fn tbl2fn3]3	0	0	[Table-fn tbl2fn2]2
Brain calcifications	[Table-fn tbl2fn1]1	0	0	[Table-fn tbl2fn1]1
Eye disorders	[Table-fn tbl2fn2]2	0	0	[Table-fn tbl2fn2]2
Intrauterine growth retardation	[Table-fn tbl2fn1]1	0	1	[Table-fn tbl2fn1]1
Microcephaly, auditory alterations and osteopathy	[Table-fn tbl2fn1]1	0	0	[Table-fn tbl2fn1]1
Hepatomegaly and jaundice	[Table-fn tbl2fn1]1	0	0	[Table-fn tbl2fn1]1
Microcephaly and ocular disorders	[Table-fn tbl2fn1]1	0	0	[Table-fn tbl2fn1]1
Microcephaly and hepatomegaly	[Table-fn tbl2fn1]1	0	[Table-fn tbl2fn1]1	[Table-fn tbl2fn1]1
Polyhydramnios	[Table-fn tbl2fn1]1	0	0	[Table-fn tbl2fn1]1
Maternal anaemia during pregnancy	[Table-fn tbl2fn1]1	0	0	0
*Total in the group of neonatal and paediatric patients*	*45/106* (***42.5%***)	*9/45* (***20.0%***)	*4/45* (***8.9%***)	*33/45* (***73.3%***)
***Total***	106	37/106 (**34.9** %)	17/106 (**16.0** %)	80/106 (**75.5** %)

*The clinical manifestations in these two positive patients included anaemia, intrauterine growth retardation and preterm delivery.

†Note the three B19V-positive cases with a fatal outcome: in two of them the foetus developed anaemia followed by hydrops and foetal death; the other was a case of spontaneous abortion.

‡One of these newborn patients had antecedents of febrile exanthema in the mother during pregnancy.

**Fig. 1.**
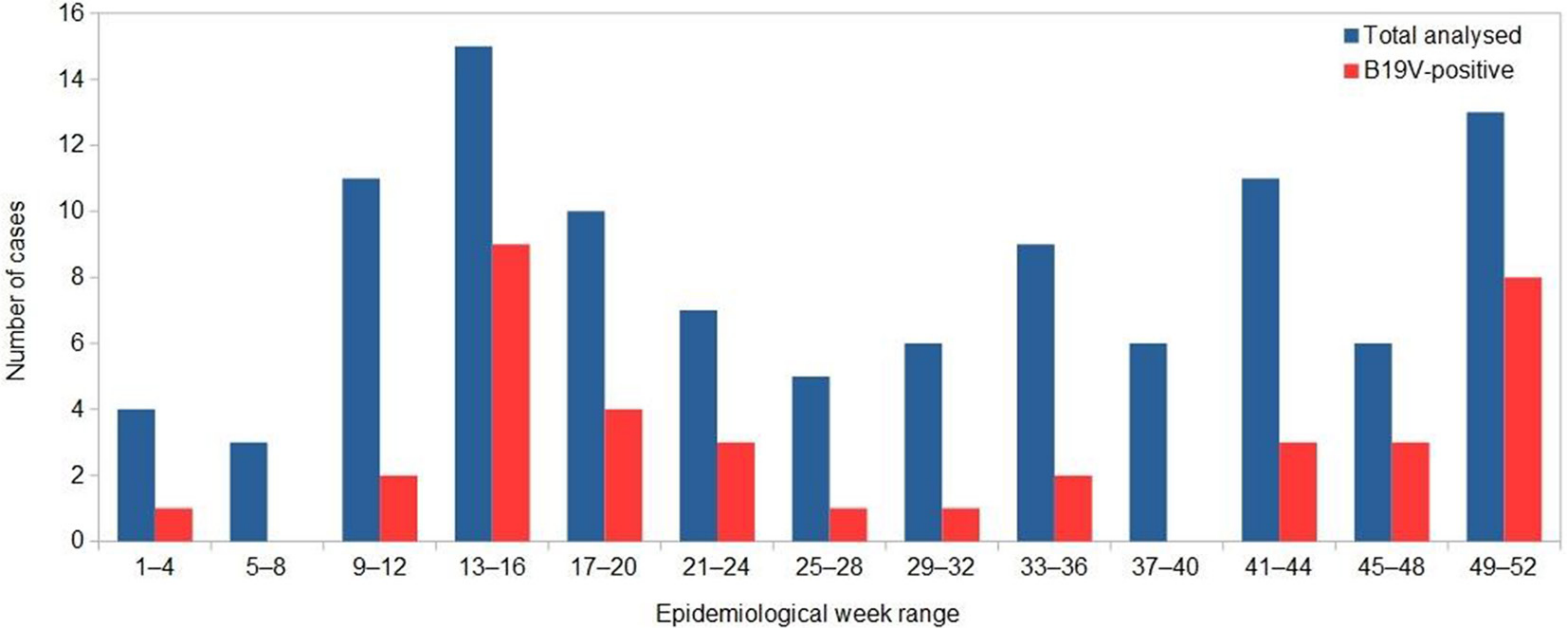
Distribution of total cases analysed and B19V-positive cases by epidemiological week range.

The distribution by epidemiological week shows the presence of the clinical situations studied throughout the year; there was a tendency for them to increase during austral autumn and spring (Fig. 1).

### Detection of human parvoviruses and associated pathologies/clinical manifestations

No sample was positive for HBoV1. In contrast, B19V DNA was detected in 37/106 samples (34.9%, 95 % CI: 26.5–44.4). Among the positive samples, 28 (75.7 %, 95 % CI: 59.9–86.6) corresponded to pregnant patients with a mean age of 25.55±6.9 years, and the remaining 9 (24.3 %, 95 % CI: 13.4–40.1) to neonatal patients (all of them less than 1 month of age). The distribution of B19V-positive cases by epidemiological week range followed the pattern of the whole sample studied (Fig. 1). Examining the results by groups of patients, the presence of B19V was detected in 28/61 (45.9 %, 95 % CI: 34.0–58.3) pregnant women and in 9/45 (20.0 %, 95 % CI: 10.9–33.8) newborns/infants (OR 3.4, 95 % CI: 1.4–8.2). The pathologies most frequently associated with the detection of B19V infection were polyhydramnios, foetal hydrops and recurrent abortion in the pregnant women, and foetal hydrops and TORCH syndrome among newborn/paediatric patients, as shown in Table 2.

### Genotyping

For genotyping, we amplified contiguous overlapping fragments of the NS1 region of B19V. The optimized protocols were applied to 10 samples selected out of the 37 B19V-positive samples on the basis of the quality/band intensity of the screening PCR product and there being sufficient sample quantity. Three out of 10 samples (2017/62, 2016/19 and 2017/16) yielded sufficient PCR product for all 4 NS1 fragments and were sequenced (Table 3) and deposited in GenBank under accession numbers MK097257–MK097259.

**Table 3.** PCR outcome for 4 overlapping fragments of the B19V NS1 region applied to 10 serum samples

**Nucleotide position*^a^***	**Serum sample ID**									
	2017/62	2016/19	2017/16	2017/30	2017/33	2015/29	2017/25	2016/39	2017/87	2015/80
673–1471	++	++	++	++	+	−	−	+	−	−
1355–2038	++	++	++	++	++	+	++	++	−	++
1817–2666	++	++	++	++	*	*	*	*	*	*
2545–3298	++	++	++	+	+	+	+	++	−	+

*a*, amplicon on the sequence with GeneBank accession number NC_000883.2

++, optimal quality (the electrophoresis and fluorometry tests of the products obtained showed their integrity, specificity and concentration as required for sequencing).

+, expected band size but low concentration of the PCR product.

*, extra band present.

−, absence of band of expected size.

**Fig. 2.**
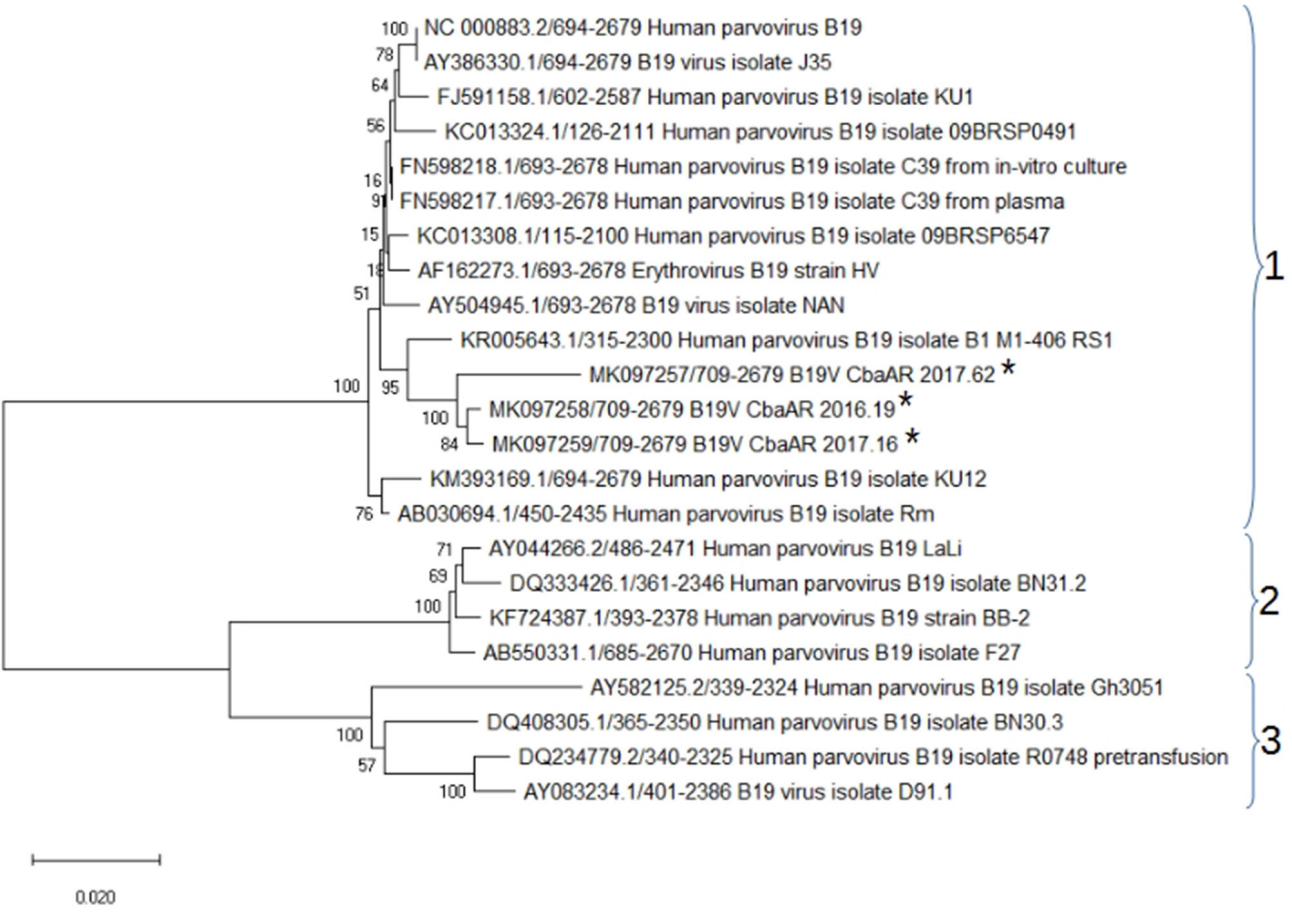
Genetic relationship between the 23 B19V NS1 sequences analysed, including the 3 local isolates (*), MK097257, MK097258 and MK097259. The tree was inferred using the neighbour-joining method. The percentage of trees in the associated taxa that grouped together (1000 bootstrap repetitions) is shown next to the branches. Reproduced to scale, with branch lengths in the same units as the distances used to infer the phylogeny. Evolutionary distances were calculated using the Kimura two-parameter model and are in the units of the number of base substitutions per site. The analysis was performed using mega x.

Genetic inference showed that all of the B19V viruses sequenced from the three samples clustered in genotype 1 (Fig. 2). The overall genetic distance among the sequences was 8.25 % and the average genetic distance within the cluster of genotype 1, including the analysed sequences, was 1.7 %.

## Discussion

The lack of detection of HBoV1 in the present study provides no indication of its presence in maternal circulation, transplacental transmission to the foetal blood and eventual foetal damage. These results complement two previous investigations in which bocavirus was not detected either, one focused on amniotic fluid samples from foetuses with hydrops or isolated effusions [27] and the other on formalin-fixed paraffin-embedded foetal tissues from cases of spontaneous and induced abortions [28]. Although they cannot be considered to be conclusive (our sample size is small and all of these studies had a retrospective, observational design), so far there is no evidence regarding potential vertical transmission of HBoV1 and foetal damage during gestation.

On the other hand, the high detection rate for B19V in the study sample [37/106 (34.9 %)] indicates substantial involvement in these clinical cases. This is in agreement with other authors [2, 29–31], who showed that B19V is a frequent aetiological agent in the above-mentioned pathologies.

B19V circulates throughout the year, with a seasonal peak in spring [1, 5, 32]. Our data reflect this trend and show the clinical impact of the infection throughout the year in the studied population.

Within the group of pregnant women, 28/61 (45.9 %) were B19V-positive, with 3 fatal outcomes; in two of these the foetus developed anaemia followed by hydrops and foetal death, while the other was a case of spontaneous abortion. As other possible causes (immune reaction, genetic failure, different agents of TORCH syndrome) were ruled out, it is possible to assign the aetiological role to B19V. In addition, the detection of B19V in three patients with recurrent abortion is striking and may justify further research.

The commonly reported global rates for vertical transmission and foetal risk are 33–35 % and 3–10 %, respectively [2, 29, 33], and it has been reported that the frequencies of hydrops and foetal death rise during epidemic outbreak periods [34–38]. However, pregnancy loss and foetal damage after B19V infection could be higher. In a survey covering 2714 obstetric centres in Japan, among which 253 reported pregnancies affected by congenital infections, 69 congenital B19V infections were confirmed. Of these, 49 (71 %) ended in spontaneous abortion and stillbirth; 17/69 (25 %) were born alive and 3 of these (18%) had hydrops, anaemia or cardiomegaly [11]. Further, B19V infection during pregnancy has also been associated with foetal echogenic bowel, abnormal brain imaging and neurodevelopmental impairment associated with high viral load [31, 39, 40], for which the virus could be underdiagnosed early in clinical practice. Therefore, it is important to maintain an updated registry of B19V circulation and to target more effort towards investigating suspected B19V infections in pregnant women. The frequency of women of childbearing age being susceptible to B19V infection is variable and may be as high as 34–55 % [41, 42], at least during interepidemic years. Thus, maternal B19V infection may be a frequent event and testing for B19V infection using complementary detection assays in clinical situations where the virus is suspected as the underlying cause is justified. Currently, differential diagnosis is reached after the recognition of the classical clinical picture in pregnant women, foetal anaemia/hydrops during prenatal ultrasound test or exposure to the virus [42]. The data from this and other studies [12, 30, 31, 39, 40] emphasize the importance of considering a wide range of markers and clinical manifestations (foetal ascites, polyhydramnios, oligohydramnios, foetal cardiac alterations and manifestations in the spectrum of TORCH, such as intrauterine growth retardation, preterm delivery, as well as the clinical presentation classically associated with B19V). It is essential to test not only specific antibodies, but also the virus DNA. The use of complementary diagnostic tools to assess B19V infection is crucial in order to offer intrauterine therapy (blood transfusion, hyper immune serum treatment) depending on the severity of the pathology, because, should it be required, it is a medical emergency and demands rapid action [29, 43]. Further, assessing B19V infection early after diagnosis will be relevant in these situations in the context of promising recent findings showing the antiviral activity of brincidofovir against B19V replication [44]⁠.

For our studied population, the data suggest a significant participation of B19V in cases of foetal hydrops as a result of congenital infection: there were 7 B19V-positive patients out of 35 with hydrops (20%), as determined from maternal or newborn samples, which is in line with the results of previous studies [2, 29, 33]⁠.

The lower frequency of detection of B19V in neonatal and paediatric patients compared to pregnant patients reflects the detected vertical transmission rate, which is commonly reported to be one-third of maternal infections in general, as mentioned above [34–38]. In agreement with that, the OR for B19V detection in pregnant patients versus newborns/infants in our series was 3.4.

Finally, three B19V genotypes have been identified to date and 5–13 % divergence among them has been reported in the nucleotide sequence [45]⁠. The prevalence of each genotype varies according to geographical location, time and clinical sample [46]⁠. The isolates identified in this study grouped in the cluster of genotype 1, which is the most prevalent genotype detected in serum worldwide [47, 48]. They represent the first B19V sequences isolated in Argentina. To date, genetic variability among B19V genotypes has not been shown to be associated with differences in biology, pathogenicity or transmission routes [49], although the registry of the types and their distribution at local and regional level, as well as their differential frequency in host tissues (blood, synovium, endothelium, myocardium), can contribute to future epidemiological studies and health surveillance [50].

In conclusion, the frequency of B19V highlights its involvement in the pathologies studied and emphasizes the importance of the molecular diagnosis of the infection in both the mother and the child. Future prospective studies with a larger sample size and a detailed registry of clinical data for the patients are justified in order to gather high-quality evidence to optimize early diagnosis in clinical practice.
